# Effects of incremental nano-selenium supplementation on growth performance, carcass traits, blood profiles, and economic efficiency in V-line rabbits

**DOI:** 10.1038/s41598-025-23823-0

**Published:** 2025-11-10

**Authors:** Ali S. El-Shafey, Ayman H. Abd El-Aziz, Saber S. Hassan, Mustafa Shukry, Nagi M. El-Shafai, Mohamed S. Moharam

**Affiliations:** 1 Department of Animal and Poultry Production, Faculty of Agriculture, Damanhour University, Damanhour, Egypt; 2 Department of Animal Wealth Development, Faculty of Veterinary Medicine, Damanhour University, Damanhour, Egypt; 3 Department of Physiology, Faculty of Veterinary Medicine, Kafrelsheikh University, Kafrelsheikh, Egypt; 4 Nanotechnology Center, Chemistry Department, Faculty of Science, Kafrelsheikh University, Kafrelsheikh, 33516 Egypt

**Keywords:** Rabbit, Nano selenium, Performance, Carcass, Haemato-biochemical, Economic efficiency, Microbial count, Biochemistry, Biological techniques, Biotechnology, Environmental sciences, Zoology

## Abstract

The use of nano-selenium (Nano-Se) in the rabbit industry has gained attention as an optimal selenium source due to its potential benefits. This study evaluated the effects of varying levels of Nano-Se in drinking water on growth, carcass traits, blood profiles, microbial counts, and economic efficiency in growing rabbits. Seventy-two male V-line rabbits (five weeks old) were divided into four groups: a control group (T1, 0 ppm Nano-Se) and three treatment groups receiving Nano-Se at 0.1 ppm (T2), 0.2 ppm (T3), and 0.3 ppm (T4) for eight weeks. Results showed significant improvements (*p* < 0.01) in growth performance, including final body weight, weight gain, feed intake, and feed conversion ratio, particularly at 0.1 ppm. Carcass yield and haemato-biochemical markers also improved significantly, with higher Nano-Se levels (0.2 and 0.3 ppm) enhancing kidney and liver functions and antioxidant activity, as reflected by reduced urea, AST, and MAD levels. Additionally, Nano-Se supplementation reduced pathogenic bacterial counts in the cecum, particularly *Escherichia coli* and *Clostridium* spp., and improved the total bacterial count at higher doses. Economically, Nano-Se supplementation decreased production costs and significantly increased net profit and efficiency, especially at the 0.1 ppm dose. In conclusion, Nano-Se, particularly at 0.1 ppm in drinking water, improved growth, health, and economic outcomes, positioning it as a valuable supplement for sustainable rabbit farming.

**Supplementary Information:**

The online version contains supplementary material available at 10.1038/s41598-025-23823-0.

## Introduction

Rabbit farming has gained significant attention recently due to their rapid growth and high reproductive rates compared to broiler chickens^1^. Rabbit meat is a nutritious, low fat and cholesterol protein source^2^. With increased rabbit production, dietary supplements have become essential for enhancing growth and health.

Rabbits provide a superior protein source for human consumption, perhaps alleviating a portion of the meat deficit in Egypt. Egypt ranks sixth in rabbit meat production, with 69.840 million tons, following China, Italy, Spain, and France (FAO 2004). Egypt’s population is undergoing significant development, prompting numerous studies to investigate new technologies to enhance feed efficiency and augment rabbits’ growth performance.

Improving animal productivity and immune responses through natural sources is a key goal in animal breeding^3^. Rabbits absorb essential nutrients and trace elements, which contribute to improved growth. Selenium (Se) plays a critical role in growth, organ function, and immune system development, protecting against pathogens. A deficiency in Se is linked to growth issues, digestive problems, and organ dysfunction. Adequate Se supplementation in rabbits promotes better health and performance, with dietary requirements ranging from 0.05 to 2.0 mg/kg depending on the diet and Se source^4^. Trace minerals like Se are vital for metabolic and physiological functions, influencing fertility, immunity, and disease prevention^5^. Se also supports antioxidant enzymes that regulate harmful byproducts of metabolism, aiding the immune system under environmental stress^6^.

The bioavailability of selenium depends on its physical form^6^. Advances in nanotechnology have brought attention to nano-selenium (Nano-Se) due to its enhanced properties, including high surface activity, catalytic efficiency, and low toxicity^7^. Nanotechnology has broad applications in agriculture and animal husbandry, improving nutrient delivery, productivity, and safety^8^. Nano-minerals require lower quantities while offering higher bioavailability and effectiveness due to their small size and large surface area^9^. Nano-Se, in particular, acts as an antioxidant, antimicrobial agent, and immune booster, enhancing growth in rabbits^4^.

Selenium is a vital trace element, supporting physiological functions such as growth, immunity, metabolism, hormone regulation, and organ health^10^. Compared to conventional Se sources, Nano-Se offers superior bioavailability, adsorption, and reduced toxicity^25^. A review of published research has demonstrated that selenium is a crucial component of the glutathione peroxidase enzyme, which functions as an antioxidant to diminish levels of hydrogen peroxide and lipid peroxides generated during normal metabolic processes. Selenium is essential for nearly all immune system activities (Surai and Kochish 2019). The existence of four selenium-containing glutathione peroxidase enzymes (GPX1, GPX2, GPX3, GPX4) in various bodily tissues consistently regulates oxidation reactions. Other enzymes, including superoxide dismutase, catalase, glutathione sulfur transferase, and the non-enzymatic substance vitamin E, contribute to preventing peroxidation processes.

Supplementing Nano-ZnO and/or Nano-Se improved growth performance, carcass yield, nutrient digestibility, and reduced fat and liver enzyme levels in rabbits^11^. Recently, adding Nano-Se, especially the level of (0.05 mg/kg), followed by organic selenium supplements in the rabbit’s diet has been beneficial in improving the production performance of rabbits, which reflects on economic efficiency compared with the control group that contains inorganic-Se^12^. According to Rajendran et al.^13^, nano-Se supplementation improved fertility, growth performance, and rumen feed efficiency. It also increases antioxidant activity enhances immunological response, gut health, and the nutritional value of animal products while having low toxicity^14^. Similarly, in fish species such as *Cirrhinus mrigala* and *Catla catla*, nano-Se supplementation significantly increased weight gain, specific growth rate, and nutrient digestibility^15^. However, lambs fed a conjugated linoleic acid (CLA)-Nano-Se mixture decreased their weights compared to the control group. Dietary nano-Se supplementation significantly affected HDL, LDL, thyroxine, triiodothyronine, and glutathione peroxidase (*P* < 0.05), but had no significant impact on glucose, triglycerides, VLDL, total protein, or cholesterol. Nano-Se supplementation at 2 mg/kg DM enhanced liver gene expression of Glutathione peroxidase 1 (GPX1) and Selenoprotein W (*P* < 0.05). Including CLA in the diet increased the peroxisome proliferator-activated receptor gamma expression and decreased stearoyl COA desaturase 1 genes in tail (*P* < 0.01), suggesting that nano-Se and CLA have not synergism interaction in the above parameters^16^. Hosseintabar-Ghasemabad^17^ reviewed Nano-Se’s benefits in broilers, showing growth and antioxidant status improvements. Nano-Se also regulates immunological responses by enhancing cytokine production and augmenting phagocytic activity. In sheep, nano-selenium improved neutrophil chemotactic and respiratory burst activity, signifying a more robust immune response relative to sodium selenite^18^. Nano-Se supplementation in fish enhanced the expression of immune-related genes, including interleukin-1β and lysozyme^19^. Optimal growth performance has been observed with Nano-Se supplementation at 0.1–0.3 mg/kg, although benefits at higher levels (up to 2–3 mg/kg) have also been reported^20^. This study investigates the effects of Nano-Se supplementation in drinking water at levels of 0.1 ppm, 0.2 ppm, and 0.3 ppm on growth performance, carcass traits, haemato-biochemical parameters, antioxidant activity, bacterial counts, and economic efficiency in V-line rabbits during the fattening stage (5–13 weeks of age) under moderate Egyptian environmental conditions.

## Materials and methods

### Study location and ethical considerations

This study, conducted at El-Bostan Poultry Farm, Faculty of Agriculture, Damanhour University, examined the effects of nano-selenium (Nano-Se) at varying levels on the productivity, physiology, carcass traits, and economic efficiency of 72 weaned V-line rabbits. All procedures were approved by the AU-IACUC at Damanhour University and were carried out in accordance with the relevant institutional guidelines and regulations, in compliance with Directive 2010/63/EU on the protection of animals used for scientific purposes. The study is reported in accordance with the ARRIVE guidelines (https://arriveguidelines.org).

### Fabrication of Se nanorod

Sodium selenite, sodium citrate, and ethanol (Sigma-Aldrich) were used as reagent-grade chemicals without further purification. To synthesize selenium nanorods, 0.02 M sodium selenite (Na₂SeO₃) was dissolved in 100 ml of double-distilled water. Gradually, 0.025 M sodium citrate was added while stirring, followed by 30 min of ultrasonication. The solution was heated for one hour to form a selenium citrate complex. Subsequently, 0.5 ml hydrazine hydrate was added dropwise to the hot mixture until it turned red. The crystalline product was centrifuged, washed with ethanol and water, and dried at 50 °C.

### Characterization techniques

The UV–vis absorption spectra were obtained using a Shimadzu UV-2450 spectrophotometer. Fourier transform infrared (FT-IR) spectra were recorded with a JASCO 4100 spectrometer using the KBr pellet method. X-ray diffraction (XRD) analysis was performed on a Shimadzu 6000 XRD diffractometer with Cu Kα radiation (λ = 1.54056 Å). Transmission electron microscopy (TEM) images were captured using a JEOL 2100 microscope at 200 kV. Zeta potential measurements were conducted with a Brookhaven zeta potential/particle size analyzer, while surface area and pore size distribution were analyzed using a BET Nova LX3 instrument.

### Experimental design, housing, and management

Seventy-two V-line rabbits were weaned at 35 days old with an average initial weight of 629.8 ± 19.6 g. The rabbit farm unit, Faculty of Agriculture, Damanhour University, provided the rabbits. Rabbits were randomly divided into four experimental groups (18 rabbits per group). Each group was split into six replicates, with three rabbits per replicate. The treatment groups included: T1 (control, no Nano-Se), T2 (0.1 ppm Nano-Se), T3 (0.2 ppm Nano-Se), and T4 (0.3 ppm Nano-Se) administered via drinking water twice weekly (Saturday and Tuesday) for eight weeks. Rabbits were housed in a naturally ventilated, east-west oriented rabbitry with galvanized wire cages (40 × 50 × 35 cm), each equipped with a metal feeder and nipple drinker. Environmental conditions, including natural ventilation and temperature, ranged between 23 and 27 °C, with 60–65% relative humidity and 14 h of daylight. Feed and water were provided *ad libitum*, and all rabbits were fed a commercial pellet diet (Table 1) containing 17.24% crude protein, 13.46% crude fiber, 2.80% fat, and 2464 kcal/kg, along with essential vitamins and minerals^21^. The formulation adhered to NRC guidelines. Rabbits had constant access to clean, fresh water. Each group was supplied with drinking water (DW) from a separate container. Water was available ad libitum for the control group T1 (control, no Nano-Se). Twice weekly (Saturday and Tuesday), for the other experimental groups T2 (0.1 ppm Nano-Se), T3 (0.2 ppm Nano-Se), and T4 (0.3 ppm Nano-Se), a fixed amount of DW (100 ml/day) was offered for each rabbit (1800 ml/treatment group) and supplied with 0.1, 0.2 and 0.3 mg Nano-Se/L of drinking water, respectively for eight weeks. In the morning, after 2 h of drinking water starvation, then fresh water was accessible ad-libitum through the rest of the day^22^.

**Table 1:** Ingredient materials and the determined analysis of the experimental diet.

Ingredients	(kg/ton)	%	Determined composition (g/kg)	
Yellow corn Barley Molasses Clover hay Wheat bran Soybean meal Dicalcium phosphate Limestone Sodium chloride Vitamin and minerals mixture* DL-methionine Total	100.0 130.0 30.0 395.0 150.0 175.0 8.0 5.0 3.0 3.0 1.0 **1000**	10 13 3 39.5 15 17.5 0.8 0.5 0.3 0.3 0.1 **100**	Dry matter Organic matter Crude protein Crude fiber Ether extract Nitrogen-free extract Ash digestible energy (kcal/kg)	903.2 804.8 172.4 134.6 28.0 569.8 95.2 2665

* The rabbit premix provided the following per 3 kg/ton of diet: vitamin A (12,000,000 IU), vitamin D3 (2,000,000 IU), vitamin E (10,000 mg), vitamin K3 (2,000 mg), vitamin B1 (1,000 mg), vitamin B2 (5,000 mg), vitamin B6 (1,500 mg), vitamin B12 (10 mg), biotin (50 mg), choline chloride (250,000 mg), pantothenic acid (10,000 mg), nicotinic acid (30,000 mg), folic acid (1,000 mg), iron (30,000 mg), copper (10,000 mg), manganese (60,000 mg), iodine (1,000 mg), selenium (100 mg), cobalt (100 mg), zinc (50,000 mg), and antioxidant (1,000 mg). Formulation followed NRC guidelines.

### Sampling

At the end of the experiment, rabbits were humanely euthanized using an intravenous overdose of sodium pentobarbital (150 mg/kg BW) following anesthesia with ketamine (35 mg/kg BW, intramuscular) and xylazine (5 mg/kg BW, intramuscular).

The rabbits were weighed weekly in the morning before feeding, with body weight gain calculated by subtracting the initial weight from the final weight divided by the total days. Weekly feed intake was recorded per rabbit, and the feed conversion ratio (FCR) was calculated as total feed intake divided by body weight gain for the same period. The performance index was determined using the formula: Performance Index = (final live body weight (kg)/FCR) × 100^23^,. Six rabbits were randomly chosen from each group at 91 days of age and slaughtered after a 12-hour fast. Using the procedures outlined, we documented the following characteristics of the carcass: pre-slaughter weight, dressing %, and organ weights^24^. At the end of the trial (13 weeks of age), blood samples were taken from six randomly selected rabbits per group. About 3 mL of blood was collected from the marginal ear vein between 8:00 and 9:00 AM using heparinized and non-heparinized vacuum tubes. Serum was obtained by centrifuging coagulated blood at 4000 rpm for 15 min and stored at −20 °C for biochemical analysis. Fresh, non-coagulated blood was immediately used for hematological analysis. Selenium content in muscle and liver was measured using an atomic absorption spectrophotometer (Buck Scientific 210 VGP, USA) following Perkin^25^and Markert^26^. Meat samples from the fore and hind limbs were deboned, and 1 g was mixed with 3 ml nitric acid and 2 ml perchloric acid, left overnight at room temperature, then heated in a 72 °C water bath for 3 h. The solution was filtered, made up to 25 ml with deionized water, and analyzed for metal concentrations.

### Hematological parameters

Hematological analysis included red blood cell (RBC) counts, hemoglobin concentration, packed cell volume (PCV), white blood cell (WBC) counts, and differential leukocyte counts. RBC counts were performed using a hemocytometer and light microscope (400x magnification)^27^. Hemoglobin concentration (g/dL) was measured with a hemoglobinometer based on^28^. PCV was determined by centrifuging blood in Wintrobe hematocrit tubes at 4000 rpm for 20 min, as described by^29,30^. WBC counts were conducted using standard methods at 100x magnification^31^, and differential leukocyte counts were prepared from Giemsa-stained blood films, following B Houwen^32^ and staining it with Giemsa. Phagocytic activity (PA) and the phagocytic index (PI) were assessed following JC Hustedt et al.^33^ Citrated blood (1 mL) was incubated with 50 µg of Candida albicans at 23–25 °C for 3–5 h, then stained with Giemsa. PA was calculated as the percentage of macrophages containing yeast cells, and PI was determined as the average number of yeast cells ingested per macrophage.

Biochemical analysis included assessments of protein, lipid profiles, kidney and liver functions, and oxidative stress markers. Serum total protein and albumin levels were determined using diagnostic kits (Diamond Diagnostics, Cairo, Egypt) and a spectrophotometer (Beckman DU-530, Germany) following methods by^34,35^. Globulin level was calculated as the difference between total protein and albumin^36^. Lipid profiles, including total lipid, triglycerides, cholesterol, HDL, and LDL concentrations, were analyzed using commercial kits^37–39^. Kidney function was evaluated by measuring serum creatinine and urea levels based on the protocols of^40,41^, Liver enzyme activities, such as alkaline phosphatase (ALP), aspartate aminotransferase (AST), and alanine aminotransferase (ALT), were assessed using methods from^42,43^. Oxidative stress markers, including total antioxidant capacity (TAC) and malondialdehyde (MDA), were evaluated using procedures outlined by^44,45^ using diagnostic kits (Diamond Diagnostics, Cairo, Egypt).

### Cecal microbiota count

The American Public Health Association states that the cecum is the best place to count the gastrointestinal tracts of healthy rabbits^46,47^. Bacteria, *Escherichia coli*, and *Clostridium* species were tested following^48^.

### Economic efficiency

Economic efficiency was calculated as the difference between total income and total costs for each experimental group^49^. Total costs included feed, rabbit purchases, and veterinary care. In contrast, total income was based on the value of the rabbits’ final live body weight (BW), using local market prices at the time of the experiment. The formulas used were:

EE (%) = (Net revenue/Total cost) × 100

REE (%) = (EE of treatment group/EE of control group) × 100

Where: Net revenue = (Price of final live BW in LE) - (Total costs in LE)

Price of final live BW (LE) = Average BW (kg/head) × Price per kg live BW (LE)

Total costs (LE) = Average cost (kg/head) × Price per kg (LE).

### Data analysis

Statistical analysis was performed on the data using the General Linear Model (GLM) function of the SAS statistical analysis system, Institute SAS^50^, utilizing the model that follows: The equation yij = µ + Ti + eij calculates the response for rabbit i under treatment j (where j = 1, 2), with ν representing the overall mean, Ti the effect of treatment j, and eij the random error term. An orthogonal contrast analysis was conducted to evaluate linear and quadratic trends across the four levels of Nano-Se supplementation. The significance of the relationship between treatment levels was determined by applying the Tukey test with a significance level of *P* < 0.05.

## Results

### Analysis of fabricated selenium (Se) nanorods

#### XRD analysis

**Fig. 1:**
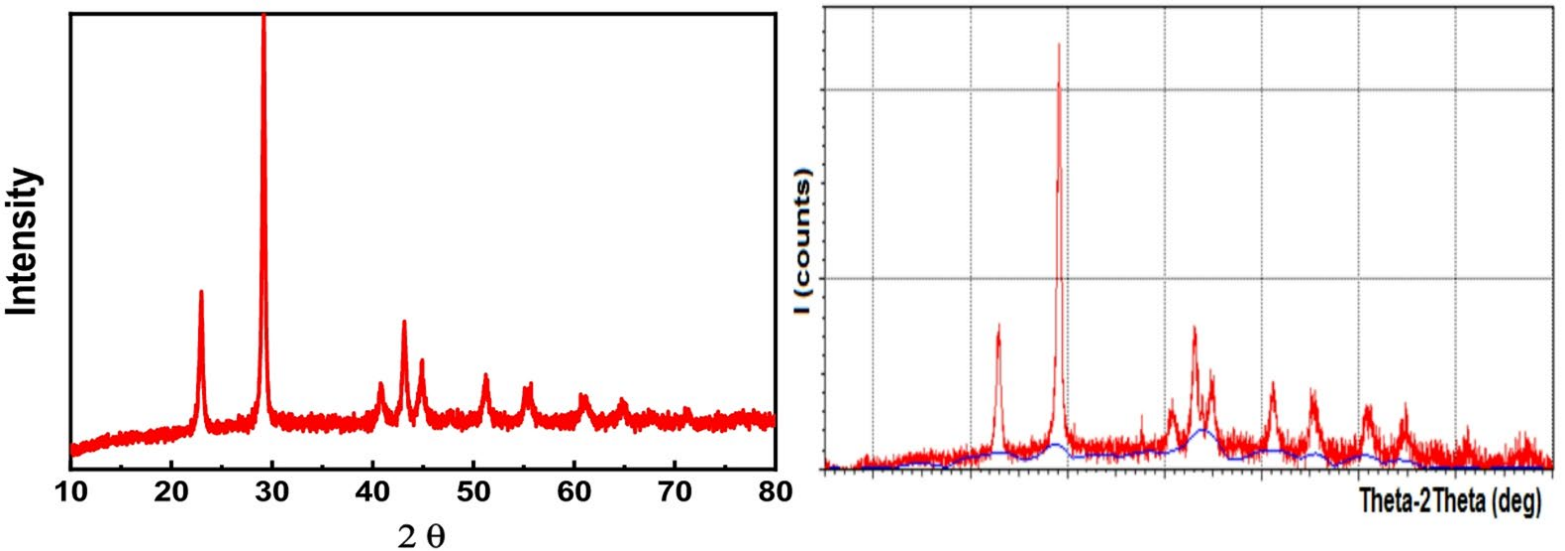
XRD pattern of Se nanorod (left) and crystallinity (right).

The XRD pattern of the synthesized Se nanorods (Fig. 1) confirms a single-phase monoclinic structure with lattice parameters: a = 4.688 Å, b = 3.423 Å, c = 5.132 Å, β = 99.506°, and V = 82.31 Å. Peaks at 23.1°, 29.2°, 40.9°, 42.8°, 44.8°, 51.1°, 55.4°, 60.8°, 65.2°, and 71.1° correspond to crystalline Se nanorods. Using Debye-Scherrer’s formula, the primary particle diameter was calculated to be 43.7 nm, and the crystallinity was found to be 60%.

#### HR-TEM analysis

**Fig. 2:**
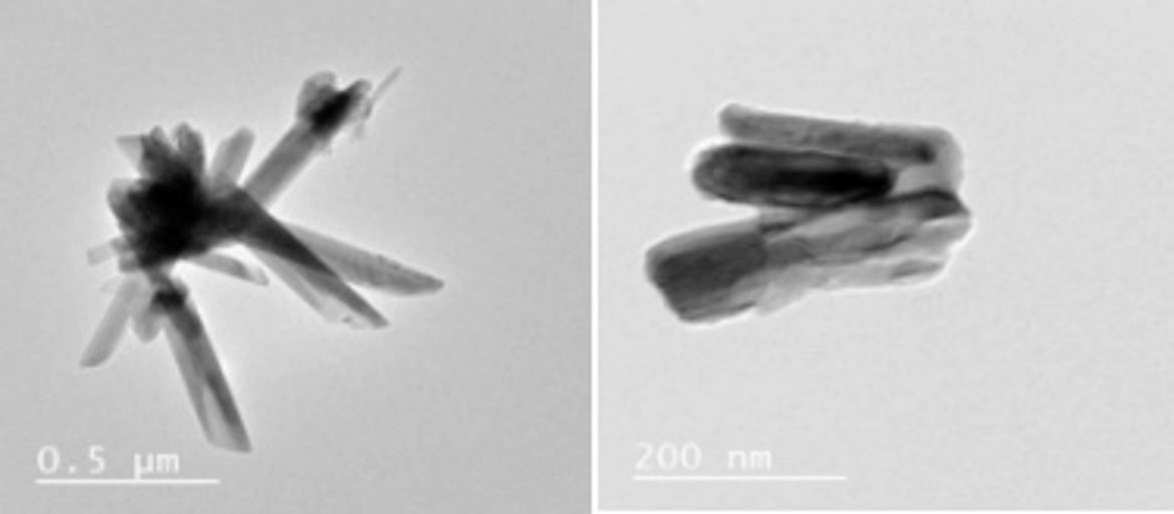
HR-TEM images of Se nanorods at different magnification.

HR-TEM images (Fig. 2) reveal uniform Se nanorods with diameters ranging from 47 to 63.6 nm and 176–200 nm lengths, consistent with XRD results. The nanostructures exhibit well-defined rod morphology.

#### UV–Vis spectroscopy

**Fig. 3:**
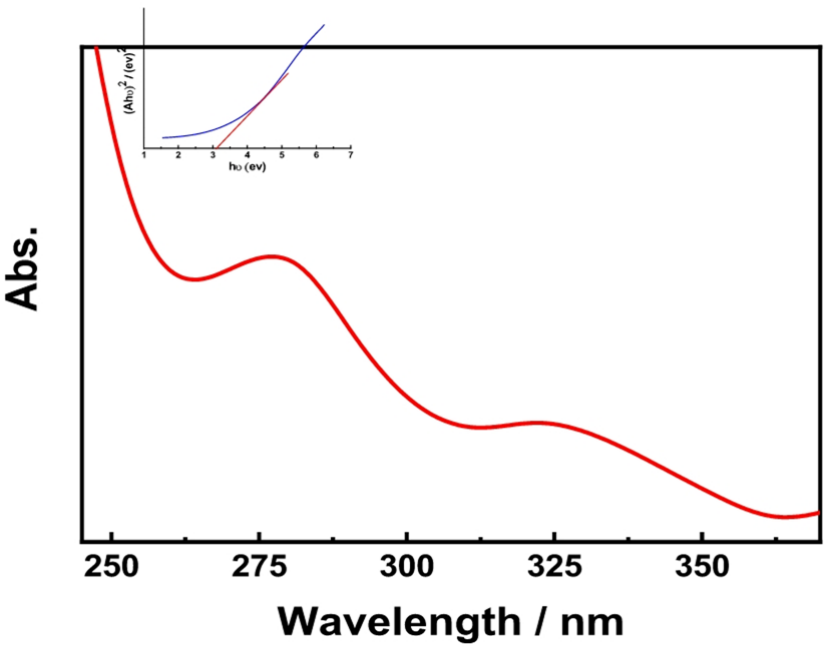
UV-visible spectra of CuO nano rods.

UV–Vis spectra of the Se nanorods, measured in double-distilled water, show a distinct absorption peak at 275 nm. The energy band gap, calculated using Tauc’s method, was 3.1 eV (Fig. 3). This band gap indicates the potential of Se nanorods for photocatalytic applications, such as solar cells and organic pollutant degradation.

#### Zeta potential analysis

**Fig. 4:**
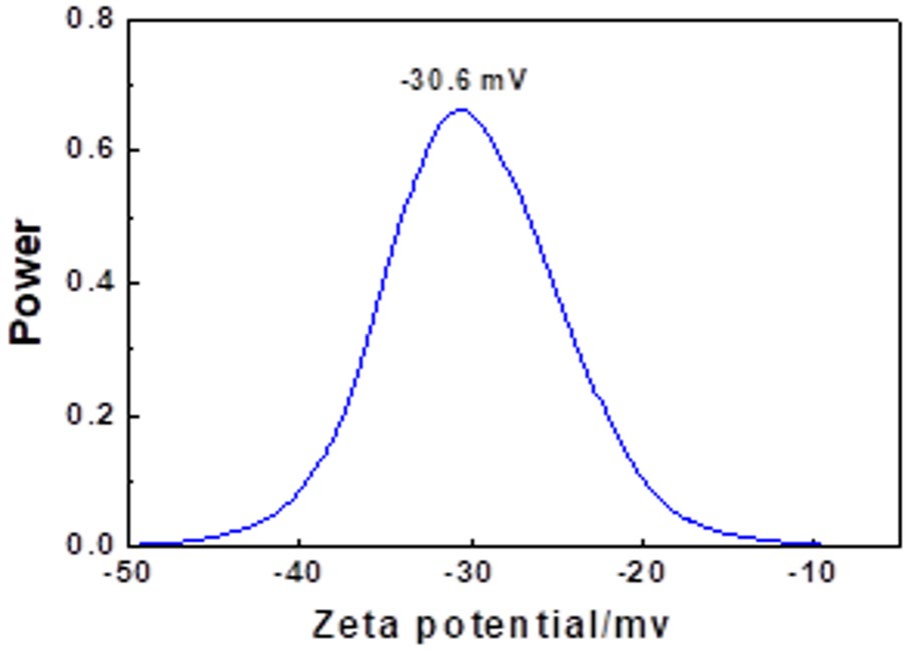
Zeta potentials of Se-nanorod in double distilled water.

The zeta potential of the Se nanorods was measured at −30.6 mV (Fig. 4), indicating good colloidal stability in the double-distilled water medium. The stability arises from the negative surface charge, preventing particle aggregation. Zeta potential values greater than ± 30 mV typically ensure stability, with additional stability provided by steric effects using nonionic surfactants or polymers. These results confirm that the Se nanorods are highly monodispersed and stable in solution under ambient conditions.

Effects of Nano-Selenium Supplementation on V-Line Rabbits.

### Growth performance (Table 2)

**Table 2:** Growth performance of V-line rabbits supplemented with varying levels of nano selenium (Nano-Se) in drinking water.

Items	Nano-Se levels (ppm)				SEM	*p*-value		
	0 ppm (T1)	0.1 ppm (T2)	0.2 ppm (T3)	0.3 ppm (T4)				
						Combined	Linear	Quadratic
Initial body weight (g)	617.22	632.22	636.11	633.89	10.05	0.908	0.551	0.67
Final body weight (g)	1743.33 ^b^	1841.25^a^	1795.71 ^ab^	1776.87 ^ab^	10.24	0.013	0.005	0.064
Body weight gain (g)	1089.17 ^b^	1191.40 ^a^	1138.90 ^ab^	1138.90 ^ab^	7.08	0.001	0.143	0.002
Total feed consumption (g)	4005.63 ^a^	3651.17 ^b^	4009.50 ^a^	3676.47 ^b^	32.99	0.001	0.046	0.873
Feed conversion ratio (g feed/g gain)	3.68 ^a^	3.07 ^d^	3.52 ^b^	3.23 ^c^	0.014	˂0.001	˂0.001	˂0.001
Performance index	47.40 ^d^	59.93 ^a^	50.93 ^c^	55.37 ^b^	0.193	˂0.001	˂0.001	˂0.001

SEM: Standard error of the means.

^a, b^ Means within a row with different superscripts are significantly different (*p* < 0.05).

The study demonstrated that initial body weight was consistent across all groups, ensuring a uniform start. However, final body weight significantly increased with Nano-Se supplementation, particularly at 0.1 ppm, which showed the highest value. Body weight gain was also considerably enhanced, with the most significant improvement observed in the 0.1 ppm group, followed by intermediate gains in higher doses, indicating a significant linear and quadratic response. Nano-Se supplementation notably reduced total feed consumption and improved feed conversion ratio (FCR), with the 0.1 ppm group achieving the most efficient feed utilization. Both linear and quadratic trends were significant for FCR and feed intake. Moreover, the performance index improved significantly with Nano-Se inclusion, peaking in the 0.1 ppm group and following a dose-responsive pattern. Overall, Nano-Se, especially at 0.1 ppm, effectively boosted growth performance, feed efficiency, and productivity in growing rabbits. Nano-Se supplementation significantly affected ashing in the fore limb, hind limb, and liver. The 0.1 ppm Nano-Se group (T2) had the highest ashing values in all tissues, followed by the 0.2 ppm (T3) and 0.3 ppm (T4) groups. The control group (0 ppm, T1) showed the lowest ashing values. Statistical analysis revealed significant differences (*p* < 0.001) across the groups, with linear and quadratic effects observed in Table S1.

### Carcass traits (Table 3)

**Table 3:** Carcass traits (%) of V-line rabbits supplemented with varying levels of nano selenium (Nano-Se) in drinking water.

Items	Nano-Se levels (ppm)				SEM	*p*-value		
	0 ppm (T1)	0.1 ppm (T2)	0.2 ppm (T3)	0.3 ppm (T4)		Combined	Linear	Quadratic
Dressing (%)	53.34 ^ab^	55.49 ^a^	51.68 ^b^	54.04^a^	0.352	0.009	0.599	0.883
Total giblets (%)	4.4	4.37	4.54	4.1	0.071	0.203	0.263	0.165
Intestine (%)	7.51 ^b^	8.3^a^	8.13 ^ab^	7.55 ^b^	0.104	0.029	0.943	0.004
Cecum (%)	8.93	7.72	8.5	8.4	0.245	0.398	0.725	0.273
Stomach (%)	1.38	1.35	1.3	1.33	0.023	0.681	0.38	0.529

SEM: Standard error of the means.

^a, b^ Means within a row with different superscripts are significantly different (*p* < 0.05).

The findings revealed that Nano-Se supplementation significantly enhanced dressing percentage, with the highest values observed at 0.1 ppm and 0.3 ppm. However, no significant linear or quadratic trends were detected. Giblet percentage remained unaffected by Nano-Se levels. Intestine percentage significantly increased, particularly in the 0.1 ppm group, with a notable quadratic trend indicating a non-linear response. In contrast, cecum and stomach percentages were not significantly influenced by treatment. Overall, Nano-Se supplementation, especially at 0.1 ppm, improved carcass yield and intestinal development without affecting other visceral components.

### Hematological parameters (Table 4)

**Table 4:** Hematology and phagocytic function in V-line rabbits supplemented with varying levels of nano selenium (Nano-Se) in drinking water.

Items	Nano-Se levels (ppm)				SEM	*p*-value		
	0 ppm (T1)	0.1 ppm (T2)	0.2 ppm (T3)	0.3 ppm (T4)				
						Combined	Linear	Quadratic
RBCs (10^6^/µl)	4.63 ^b^	5.13 ^a^	5.23 ^a^	5.23 ^a^	0.048	0.001	˂0.001	0.016
Hemoglobin (Hgb %)	10.0 ^c^	11.33 ^ab^	10.33^bc^	11.67 ^a^	0.183	0.012	0.024	1.0
Packed cell volume (PCV)	31.0 ^b^	33.33 ^ab^	31.67 ^b^	34.67 ^a^	0.438	0.032	0.027	0.707
WBCs (10^3^/µl)	6.43 ^b^	7.1 ^a^	7.1 ^a^	7.07 ^a^	0.047	˂0.001	˂0.001	0.001
Lymphocytes (10^3^/µl)	46.0	46.33	45.67	45.0	0.325	0.526	0.222	0.451
Monocytes (10^3^/µl)	15.33 ^ab^	15.0 ^b^	16.0 ^ab^	16.33^a^	0.175	0.05	0.019	0.352
Basophils	0.33 ^b^	0.33 ^b^	1.0 ^a^	0.0 ^b^	0.075	0.001	0.623	0.003
Eosinophils	12.0	12.0	12.67	11.67	0.217	0.444	0.866	0.264
Neutrophils	26.33	26.33	24.67	27.0	0.447	0.322	0.934	0.207
**Phagocytic function**								
Phagocytic activity (PA)	20.0	19.67	21.0	19.33	0.217	0.07	0.753	0.141
Phagocytic index (PI)	17.33	16.33	16.67	17.0	0.204	0.368	0.719	0.118

SEM: Standard error of the means.

^a, b^ Means within a row with different superscripts are significantly different (*p* < 0.05).

Nano-Se supplementation significantly improved several hematological parameters in growing rabbits. RBC count, hemoglobin concentration, and packed cell volume notably increased, particularly in higher-dose groups, showing linear and quadratic trends. Total WBC count also rose significantly across all supplemented groups, indicating enhanced immune status. While lymphocyte levels remained unaffected, monocyte and basophil counts significantly increased, especially at higher Nano-Se levels. Eosinophil and neutrophil counts were not influenced by treatment. However, phagocytic activity and index showed no statistical differences, and slight numerical improvements were observed. Nano-Se supplementation (0.1–0.3 ppm) positively influenced hematopoietic and immune-related blood indices.

### Biochemical traits (Table 5)

**Table 5:** Biochemical parameters of V-line rabbits supplemented with varying levels of nano selenium (Nano-Se) in drinking water.

Item	Nano-Se levels (ppm)				SEM	*p*-value		
	0 ppm (T1)	0.1 ppm (T2)	0.2 ppm (T3)	03 ppm (T4)				
						Combined	Linear	Quadratic
Protein profile								
Total Protein (g/dl)	5.93 ^c^	6.23 ^ab^	6.06^bc^	6.40 ^a^	0.033	˂0.001	˂0.001	0.803
Albumin (g/dl)	2.7 ^b^	2.83 ^b^	2.8^b^	3.07 ^a^	0.031	0.004	0.001	0.298
Globulin (g/dl)	3.23	3.4	3.27	3.33	0.032	0.282	0.562	0.438
Lipid profile								
Total lipids (mg/dl)	212.67 ^a^	209.0 ^b^	211.0 ^ab^	211.67 ^a^	0.384	0.021	0.774	0.01
Triglycerides (mg/dl)	116.33 ^a^	110.33 ^c^	112.67 ^b^	113.0 ^b^	0.274	˂0.001	0.005	˂0.001
Cholesterol (mg/dl)	97.0 ^a^	92.0^b^	93.0 ^b^	92.33 ^b^	0.391	0.001	0.001	0.021
HDL	6.30	6.40	6.40	6.53	0.037	0.203	0.047	0.824
LDL	15.33	13.67	13.0	11.33	0.671	0.239	0.048	1.0
Kidney function test								
Urea (mg/dl)	25.33 ^a^	24.67 ^a^	21.0 ^b^	24.67 ^a^	0.387	0.003	0.118	0.011
Creatinine (mg/dl)	1.13	1.17	1.2	1.13	0.024	0.729	0.879	0.313
Liver function test								
ALT (U/L)	31.0	29.0	29.0	30.0	0.398	0.259	0.409	0.079
AST (U/L)	18.33 ^a^	18.33 ^a^	17.0 ^ab^	16.0 ^b^	0.298	0.031	0.005	0.412
Alkaline phosphatase	77.33	77.67	76.33	76.67	0.23	0.188	0.12	1.0
Antioxidant enzymes								
TAC (mmol/L)	146.33	147.67	152.33	149.33	0.972	0.185	0.132	0.278
MAD (nmol/ml)	2.03 ^a^	1.9 ^ab^	1.77 ^b^	1.77 ^b^	0.032	0.021	0.642	0.005

SEM: Standard error of the means. ^a, b^ Means within a row with different superscripts are significantly different (*p* < 0.05). HDL: high-density lipoprotein, LDL: low-density lipoprotein; AST: aspartate aminotransferase, ALT; alanine aminotransferase, TAC: total antioxidant capacity; MAD: malondialdehyde.

Nano-Se supplementation significantly enhanced serum biochemical parameters in growing rabbits. Total serum protein and albumin concentrations increased, particularly at higher supplementation levels, following a clear dose-dependent pattern, while globulin remained unchanged. Lipid metabolism positively modulated triglycerides and cholesterol levels significantly decreased across all Nano-Se groups, with strong linear and quadratic trends. Although HDL and LDL showed favorable trends, the differences were not statistically significant. Urea levels significantly declined, especially at 0.2 ppm, while creatinine remained unaffected. Liver enzyme AST was reduced at 0.3 ppm, indicating a potential hepatoprotective effect, whereas ALT and alkaline phosphatase were unchanged. Antioxidant status improved, with reduced MDA levels indicating lower lipid peroxidation, although total antioxidant capacity showed no significant differences. Overall, Nano-Se supplementation, particularly at 0.2–0.3 ppm, beneficially influenced protein and lipid metabolism, kidney and liver function, and oxidative stress markers in V-line rabbits.

### Gut microbiota and antibacterial effects (Table 6)

**Table 6:** Cecal bacterial counts (Log10 CFU g − 1) of V-line rabbits supplemented with varying levels of nano selenium (Nano-Se) in drinking water.

Items	Nano-Se levels (ppm)				SEM	*p*-value		
	0 ppm (T1)	0.1 ppm (T2)	0.2 ppm (T3)	0.3 ppm (T4)				
						Combined	Linear	Quadratic
Total bacterial count (TBC)	3.17 ^a^	3.0 ^a^	2.97 ^a^	2.0 ^b^	0.055	˂0.001	˂0.001	0.002
*Bacilli*	0.6 ^c^	0.77 ^a^	0.67^bc^	0.7 ^ab^	0.012	0.001	0.072	0.01
*Escherichia coli* (E. coli)	1.17 ^a^	0.97 ^c^	0.9 ^c^	1.07 ^b^	0.016	˂0.001	0.017	˂0.001
*Clostridium* spp.	0.87 ^a^	0.5 ^c^	0.7 ^b^	0.80 ^a^	0.017	˂0.001	1.0	˂0.001

SEM: Standard error of the means.

^a, b^ Means within a row with different superscripts are significantly different (*p* < 0.05).

Nano-Se supplementation significantly reduced total bacterial count (TBC), with the lowest values recorded at 0.3 ppm, indicating a strong dose-dependent antimicrobial effect. Beneficial Bacilli populations were increased considerably, particularly at 0.1 ppm, suggesting selective support of beneficial gut microbes. In contrast, pathogenic bacteria such as *E. coli* and *Clostridium* spp. were markedly decreased in all Nano-Se-treated groups, with the most notable reductions at 0.1–0.2 ppm. These effects followed both linear and quadratic trends. Overall, Nano-Se supplementation improved gut microbial balance by suppressing harmful bacteria and promoting beneficial ones, enhancing intestinal health in rabbits.

### Economic efficiency (Table 7)

**Table 7:** Economic efficiency of V-line rabbits supplemented with varying levels of nano selenium (Nano-Se) in drinking water.

Items	Nano-Se levels (ppm)				SEM	*p*-value		
	0 ppm (T1)	0.1 ppm (T2)	0.2 ppm (T3)	0.3 ppm (T4)				
						Combined	Linear	Quadratic
TBW (g)	1.743 ^b^	1.836 ^a^	1.792 ^ab^	1.787 ^ab^	0.01	0.037	0.361	0.027
Total Revenue	78.47 ^b^	82.63 ^a^	80.63 ^ab^	80.4 ^ab^	0.461	0.037	0.368	0.027
Total Feed Cost	22.43 ^a^	20.43 ^b^	22.47 ^a^	20.6 ^b^	0.183	˂0.001	0.047	0.857
Total Cost	72.43 ^a^	70.47 ^b^	72.47 ^a^	70.67 ^b^	0.184	0.001	0.058	0.823
Net Revenue	6.02 ^c^	12.14 ^a^	8.11 ^b^	9.75 ^b^	0.353	˂0.001	0.035	0.005
Economic Efficiency (EE)	8.31 ^c^	17.22 ^a^	11.18 ^b^	13.79 ^b^	0.48	˂0.001	0.025	0.004
Relative Economic Efficiency (REE)	100.0 ^d^	207.23 ^a^	134.47 ^c^	165.96 ^b^	5.078	˂0.001	0.012	0.001

SEM: Standard error of the means.

^a, b^ Means within a row with different superscripts are significantly different (*p* < 0.05).

Nano-Se supplementation significantly enhanced total body weight and revenue, with the highest values observed in the 0.1 ppm group, reflecting improved market performance. A significant quadratic trend supported these findings. Additionally, total feed and production costs were significantly reduced, especially at 0.1 and 0.3 ppm, demonstrating the cost-saving potential of Nano-Se. Net revenue increased markedly in all supplemented groups, with the 0.1 ppm group achieving the highest profitability. Economic efficiency (EE) and relative economic efficiency (REE) were significantly improved, peaking at 0.1 ppm. Overall, Nano-Se supplementation, particularly at 0.1 ppm, proved economically beneficial by enhancing growth performance, improving feed efficiency, and reducing production costs in rabbit farming.

## Discussion

Nano-selenium (Nano-Se) has garnered attention for its high bioavailability, catalytic activity, and reduced toxicity compared to conventional selenium sources^51^. Nano-selenium (Nano-Se) is recognized for its superior bioavailability, catalytic activity, and lower toxicity than conventional selenium sources^51^. In this study, Nano-Se supplementation significantly enhanced growth performance metrics such as final body weight (BW), daily body weight gain (BWG), feed conversion ratio (FCR), and the performance index, particularly at a low dose of 0.1 ppm. This result may be seen as a reaction to enhanced bioavailability, specific surface area, surface activity, catalytic efficiency, and robust adsorption capacity at various concentrations of nano-Se. This resulted in an enhancement of overall digestibility (%) and nutritional values (%), which was evident in the rise of average live body weight (LBW) and daily gain performance. Moreover, enhanced gut health presumably facilitates nutrition absorption and immunological function, which is essential for general health and productivity in rabbits^12^. Sheiha et al.^52^. reported that dietary supplementation with Nano-Se resulted in greater weights than selenium selenite and the control group without supplemented Se, attributed to superior absorption and enhanced bioavailability of Nano-Se. Similarly, Diana et al.^53^ found that rabbits’ growth performance improved by including Nano-Se additions in their diets. These findings align with previous research indicating that Nano-Se improves growth performance in rabbits and broilers^11,54^. For example^52^, observed improved BW and FCR at 25 and 50 mg/kg Nano-Se doses. Similarly, E-D Mahmoud H, D Ijiri, TA Ebeid and A Ohtsuka^55^ reported increased BW and weight gain in broilers fed 0.3 mg/kg Nano-Se, and^56^ found similar results with doses of 1–3 mg/kg. The enhanced bioavailability of Nano-Se allows for smaller doses to achieve more significant effects, making it a cost-effective growth promoter^57–59^. Selenium’s role as an antioxidant and regulator of immune and metabolic functions likely underpins these benefits^60,61^. Our results showed that supplementation increased selenium accumulation in rabbit tissues, consistent with previous studies reporting higher selenium concentrations in animal tissues^62^. 140 g of selenium-enriched rabbit meat could meet the daily selenium requirements for adults^63^. These improvements can be attributed to the enhanced bioavailability of nano-selenium, which is more efficiently absorbed and utilized by the body compared to traditional selenium sources. This increased bioavailability likely contributes to a greater activation of antioxidant enzymes, leading to reduced oxidative stress, which is known to improve overall health and performance^64^.

In poultry, three principal categories of selenoproteins influence physiological function: thioredoxin reductases (TrxR), iodothyronine diiodinases, and glutathione peroxidases (GSH-Px). TrxR comprises thioredoxin (Trx) and NADPH, which influence the cellular redox system. Concurrently, it plays a pivotal role in essential processes such as DNA synthesis, bolstering the body’s defense against oxidative stress, maintaining endoplasmic reticulum integrity, synthesizing thyroid hormones, and regulating gene expression of transcription factors, including (NF-ƙB), apurinic/apyrimidinic endonuclease/redox factor-1 (Ref-1), activator protein-1 (AP-1), apoptosis-regulating kinase (ASK1), and glucocorticoid receptor^65^. This effects collection influences health, immunological responses, and total bodily growth^65^. In poultry, nanoparticles serve various physiological functions, whether as nutritional or non-nutritive additives. Notably, they enhance vital biological reactions by elevating compound levels, facilitate enzymatic reactions by prolonging shelf life in the digestive tract, optimize digestion and absorption processes, reduce excretion, and assist in the translocation of substances through capillaries for effective cellular absorption^17^. In addition, Nano-Se and CLA had distinct effects on gene expression in the liver and tail while positively influencing specific blood parameters^16^.

Nano-Se supplementation significantly improved carcass traits, with higher dressing percentages observed in groups receiving 0.1 ppm and 0.3 ppm Nano-Se. This is due to an increase in both live body weight and average daily gain, which was reflected in an improvement in the different carcass traits^12^. The current findings align with those of^52^, who indicated that the inclusion of Nano-Se synthesized via a biological method (BIO25 and BIO50, corresponding to 25 and 50 mg of nano-Se/kg diet, respectively) in the diet of growing rabbits at 50 mg/kg significantly enhanced growth performance and carcass characteristics compared to the control group. These improvements align with findings by^11^, who reported enhanced dressing percentage and reduced fat-BW ratios with Nano-Se. Improved nutrient digestion and reduced abdominal fat likely contribute to these results^66^. Similarly, Nano-Se enhanced organ weights such as liver, kidneys, and spleen in rabbits^67^. However, some studies found no significant effects on carcass traits in broilers, highlighting the variability across species and experimental conditions^68^.

Nano-Se supplementation improved hematological parameters, significantly increasing WBCs, RBCs, hemoglobin, and packed cell volume (PCV), particularly at 0.3 ppm. Enhanced protein metabolism was evident through increased serum total protein and albumin levels, while lipid profiles showed reduced triglycerides, cholesterol, and total lipids^69^. It was documented that enhancing humoral and cellular immunological responses by elevating GSH-Px, superoxide dismutase, and red blood cell catalase levels in broiler chickens and laying hens following nano selenium supplementation was promising^70^. These findings underscore selenium’s role in lipid metabolism and antioxidant defense, reducing oxidative stress markers like malondialdehyde (MDA) and improving immune responses^71^. The high bioavailability is the primary driver of research on nanotechnology in poultry nutrition, which frequently yields encouraging and less dangerous results for nanomaterials. These additives, which come in protein capsules, nanoemulsions, and micelles, are helpful as growth promoters and immune modulators^72^. The results are consistent with those of Ibrahim et al.^68^, who observed similar hematological improvements in broilers supplemented with Nano-Se.

Furthermore, selenium enhances the activity of key selenoenzymes, such as glutathione peroxidase, contributing to cellular protection^52^. This aligns with previous studies where selenium supplementation increased antioxidant defense and protects **against cellular damage**^73^. Specifically, **due to its small particle size and high surface area**,** nano-selenium** may enhance the absorption of selenium, allowing for more efficient incorporation into the body’s **antioxidant systems**, such as **glutathione peroxidase** and **superoxide dismutase**^64^. These enzymes are crucial in neutralizing harmful free radicals, which could explain the improvements in **hematological parameters** and **overall health** observed in the rabbits.

Nano-Se also modulates immune responses by up-regulating cytokines and improving phagocytic activity. For example, in sheep, nano-Se enhanced the chemotactic and respiratory burst activities of neutrophils, indicating a stronger immune response than sodium selenite^74^. In fish, nano-Se supplementation increased the expression of immune-related genes such as interleukin-1β and lysozyme^19^. However, Selim et al.^75^ observed that plasma total proteins, albumin, globulin, the albumin/globulin ratio, and creatinine concentration were not substantially influenced by Nano-Se supplementation in broiler diets. Qin et al.^76^determined that the concentrations of blood total protein, cholesterol, HDL, triglycerides, ALT, and AST were not substantially influenced by supplementation with Nano-Se or sodium selenite in growing rabbits.

Nano-Se supplementation improved gut health by reducing harmful bacteria (*E. coli*, *Clostridium spp.*) and increasing beneficial bacteria (Bacilli) in the gut microbiome, particularly at 0.1 ppm and 0.3 ppm. Previous studies indicated that enhancing intestinal health by increasing the prevalence of beneficial microbes (*Lactobacillus* and *Faecalibacterium*) and promoting the production of short-chain fatty acids following the consumption of selenium-containing nanoparticles may represent a viable application of nano-additives^72^. The primary objective of employing nanoparticles in poultry nutrition is to diminish pathogenic bacteria, hence enhancing growth performance through the promotion of beneficial bacteria, modification of the ecology, and provision of nutrients for the advantageous microbiota within the digestive system. Numerous nano additives enhance feed efficiency by diminishing bacterial toxin production, optimizing nutrient absorption via intestinal epithelium thinning, and decreasing the circulation and mobility of intestinal mucosa epithelial cells, thereby augmenting the birds’ nutrient utilization^77^. These findings are consistent with research showing Nano-Se’s antibacterial properties and ability to enhance gut microbiota and short-chain fatty acid production^78^. Improved gut health likely supports nutrient absorption and immune function, which are critical for overall health and productivity in rabbits^12^. Selenium’s antioxidant properties contribute to gut tissue integrity and microbiome balance, promoting health and nutrient efficiency. The antimicrobial properties of selenium, particularly in its nano-form, are well-documented, and our results support the notion that selenium supplementation may have immune-modulating effects, thereby enhancing the immune response and promoting a healthier gut microbiome^79^. Nano-selenium’s ability to influence the gut microbiota could be linked to its antioxidant activity, which can support the intestinal mucosal barrier and enhance resistance to pathogens^80^.

Nano-Se supplementation significantly improved the economic efficiency of rabbit production, with the 0.1 ppm group achieving the highest net revenue and lowest feed costs (*P* < 0.001). These results align with^12^, who demonstrated that Nano-Se is a cost-effective alternative to other selenium sources, enhancing profitability through better growth performance and carcass yield. The reduction in feed costs per unit of weight gain and improved marketable weights underscore the economic advantages of Nano-Se for sustainable production. Nano-Se supplementation, particularly at 0.1 ppm, enhances growth performance, carcass traits, blood health, gut microbiota, and economic efficiency in rabbits. Similarly, Hu et al.^81^, determined that Nano-Se was more cost-effective than the control group. Economically, the nano-selenium supplementation, especially at a dose of 0.1 ppm, significantly increased the net profit and economic efficiency, making it a promising, cost-effective supplementation strategy for sustainable rabbit farming. The reduction in feed costs per unit of weight gain and improvements in carcass yield suggest that nano-selenium can optimize both performance and profitability. This highlights its potential as a valuable addition to rabbit production systems, mainly where cost efficiency is a priority^82^.

These findings highlight Nano-Se’s potential as an effective and sustainable supplement in rabbit production. Further research could optimize its application across different conditions and production systems. Future studies should explore the long-term effects of nano-selenium supplementation, particularly its impact on reproductive performance and longevity in rabbits. Additionally, optimal dosage levels for various breeds and production systems should be explored to refine their practical applications. Understanding how nano-selenium influences immune function and gut microbiota will be crucial in further establishing its role in sustainable animal husbandry.

While the study highlights the significant benefits of nano-selenium (Nano-Se) supplementation in V-line rabbits, there are a few limitations to consider. The optimal dose was identified as 0.1 ppm, but the non-linear responses observed at higher doses (0.2 and 0.3 ppm) suggest that further research is needed to refine the dosage range. Additionally, the study’s short duration (8 weeks) limits understanding of the long-term effects of Nano-Se, particularly concerning selenium accumulation and potential toxicity. The findings were based on controlled environmental conditions, and the results may not fully apply to different climates or production systems, so broader testing is necessary. Furthermore, while the study observed reduced harmful gut bacteria, more detailed microbiome analyses would help clarify the underlying mechanisms. Despite these limitations, the study’s strengths include its innovative approach of administering Nano-Se through drinking water, providing a more bioavailable form of selenium. The comprehensive assessment of various parameters—growth, health, biochemical traits, and economic efficiency—adds to the robustness of the research. Moreover, the financial benefits of Nano-Se, including reduced feed costs and increased profitability, make it a valuable tool for sustainable rabbit farming. Overall, Nano-Se supplementation at 0.1 ppm offers promising results for improving productivity and health in rabbits. However, further studies are needed to explore the long-term effects, optimal doses, and mechanisms involved.

## Conclusions

In conclusion, supplementing nano-selenium (Nano-Se) in drinking water for growing V-line rabbits significantly enhanced their growth performance, health status, and economic efficiency. The optimal dose of Nano-Se, particularly at 0.1 ppm, resulted in the best improvements in body weight gain, feed conversion ratio, and performance index. Additionally, Nano-Se supplementation improved carcass yield, blood profiles, and antioxidant activity while reducing pathogenic microbial counts in the cecum, suggesting enhanced gut health and immune function. The economic analysis further supports Nano-Se’s potential as a cost-effective supplement, demonstrating increased net revenue and economic efficiency, especially at the 0.1 ppm level. These findings highlight Nano-Se as a promising, sustainable, and bioavailable supplement that could benefit the rabbit farming industry, offering a pathway to improved productivity and profitability. Future research should explore long-term effects and refine the optimal dosage across various production systems to maximize the benefits of Nano-Se supplementation in rabbit farming. This study is the first to investigate the effects of nano-selenium supplementation via drinking water on various health and performance parameters in rabbits. While previous research has focused on selenium through feed, there has been limited exploration of nano-selenium in drinking water.

Additionally, most studies have not comprehensively assessed its impact on blood profiles, carcass traits, microbial counts, and economic efficiency. Our work fills this gap by evaluating the effects of different nano-selenium doses (0.1, 0.2, and 0.3 ppm). It provides novel insights into its potential as a bioavailable and cost-effective supplement for improving rabbit productivity and health. Further studies are required to determine the optimal safety limits of Nano Se supplementation to improve both productive and reproductive performance in rabbits and other livestock while preventing toxicity or hyperaccumulation in animal tissues.

## Supplementary Information

Below is the link to the electronic supplementary material.

Supplementary Material 1

## Data Availability

Upon request from the corresponding authors.
