# Supplementary material for: Effects of incremental nano-selenium supplementation on growth performance, carcass traits, blood profiles, and economic efficiency in V-line rabbits
Source: Sci Rep. 2025 Nov 10;15:39374. doi: 10.1038/s41598-025-23823-0 (PMC12603265; doi:10.1038/s41598-025-23823-0)
Supplement: Supplementary file 1 — Supplementary Material 1 [file 41598_2025_23823_MOESM1_ESM.pdf]

Table S1: Selenium content in the muscle and the liver of the V-line rabbits supplemented with varying levels of nanoselenium (Nano-Se) in drinking water

**Items**	**Nano-Se levels (ppm)**				**SEM**	**p-value**		
	0 ppm (T1)	0.1 ppm (T2)	0.2 ppm (T3)	0.3 ppm (T4)		Combined	Linear	Quadratic
**Forelimb ashing**	6.03^{c}	8.22^{a}	7.42^{ab}	6.48^{b}	0.33	<0.001	0.014	0.002
**Hind limb ashing**	6.37^{c}	8.11^{a}	7.01^{ab}	6.48^{b}	0.48	<0.001	0.012	0.011
**Liver ashing**	6.23^{c}	7.45^{a}	6.77^{ab}	6.25^{b}	0.26	<0.001	0.016	0.013
